# Association between iron deficiency anemia and sleep duration in the first year of life

**DOI:** 10.1590/1984-0462/2024/42/2022173

**Published:** 2023-07-24

**Authors:** José Israel Rodrigues, Victória Gabriella Fidelix de Mecenas, Márcia de Oliveira Lima, Risia Cristina Egito de Menezes, Priscilla Márcia Bezerra de Oliveira, Giovana Longo-Silva

**Affiliations:** I Universidade Federal de Alagoas, Maceió (AL), Brazil.

**Keywords:** Anemia, iron-deficiency, Sleep, Sleep deprivation, Child, Anemia ferropriva, Sono, Privação do sono, Criança

## Abstract

**Objective:**

The aim of this study was to investigate the association between iron deficiency anemia and sleep duration in the first year of life.

**Methods:**

A total of 123 infants were investigated, with sleep being evaluated at 3, 6, and 12 months of age and anemia at birth and 6 months. The cutoff points for anemia and short sleep duration were hemoglobin <11 g/dL (at birth and/or 6 months) and <10 h (at 3, 6, and 12 months), respectively. The comparison of the average sleep time between infants with and without anemia was performed using the Student’s t-test, and logistic regression models were also used to verify differences in the sleep duration (short/not short) between the groups. Linear regression analyses were conducted to determine the association between sleep duration and hemoglobin values. The analyses were adjusted for potential confounders.

**Results:**

Children with anemia were more likely to be short sleepers [odds ratio (95% confidence interval (CI)): 4.02 (1.02–15.76); p≤0.05], and for each unit increase in hemoglobin values, the sleep duration increased by 16.2 min [β (95%CI): 0.27 (0.00–0.55); p≤0.05), regardless of family income, maternal schooling, gender, and body mass index at birth.

**Conclusions:**

Our results suggest that iron deficiency anemia is associated with short sleep duration in the first year of life and indicate the need for longitudinal investigations, with longer follow-up, to verify the impact of anemia on sleep duration at subsequent ages.

## RESUMO

**Objetivo:**

Investigar a associação entre a anemia por deficiência de ferro e a duração do sono no primeiro ano de vida.

**Métodos:**

Foram avaliadas 123 crianças, sendo o sono investigado aos três, seis e 12 meses de idade e a anemia ao nascimento e aos seis meses. Utilizaram-se como pontos de corte para anemia e curta duração de sono, respectivamente, hemoglobina<11 g/dL (nascimento e/ou seis meses) e tempo total <10 h (3, 6 e/ou 12 meses). A comparação do tempo médio de sono entre as crianças com e sem anemia foi realizada pelo teste *t* de Student e modelos de regressão logística foram usados para verificar diferenças na duração do sono (curta/não curta) entre os grupos. Análises de regressão linear foram conduzidas para determinar a associação entre a duração do sono e valores de hemoglobina. As análises foram ajustadas para potenciais confundidores.

**Resultados:**

As crianças com anemia tiveram maior chance de apresentar curta duração do sono [*odds ratio* — OR (intervalo de confiança — IC95%): 4,02 (1,02–15,76); p≤0,05]. Para cada unidade de aumento nos valores da hemoglobina, o tempo de sono aumentou em 16,2 min [β (IC95%): 0,27 (0,00–0,55); p≤0,05), independentemente de renda familiar, escolaridade materna, sexo e índice de massa corporal ao nascimento.

**Conclusões:**

Nossos resultados sugerem que a anemia ferropriva está associada à curta duração do sono no primeiro ano de vida e indicam a necessidade de investigações longitudinais, com maior tempo de seguimento, para verificar o impacto da anemia na duração do sono em idades subsequentes.

## INTRODUCTION

In the first years of life, sleep undergoes expressive changes and acquires special importance due to its relationship with growth and development mechanisms. Sleep duration is a frequently investigated sleep measure concerning health outcomes, and many studies have shown that adequate sleep duration is associated with better attention, behavior, cognitive functioning, emotional regulation, and physical health among children.^1,2,3^ Therefore, the American Academy of Sleep Medicine provides age-specific sleep duration recommendations to promote optimal health, considering a total sleep time of fewer than 10 h in the first year of life inappropriate.^1^

According to the National Survey of Children’s Health data, during 2016–2018, approximately one-third of individuals aged 4 months to 17 years (34.9%) got less sleep than is recommended for their age.^4^ Although environmental and behavioral factors, such as excessive nocturnal exposure to artificial light and lack of a sleep routine established by parents, are relevant aspects of child sleep quality, iron deficiency anemia has recently been studied as one of the clinical conditions that could impact sleep duration, with effects that can last into later ages.^5–7^

However, such studies are mostly with adults and are cross-sectional, and the results remain controversial and without consensus regarding the existence of the association between the variables, as well as the direction of this association. While some studies hypothesize that anemia influences sleep duration,^5–7^ others identify sleep duration as a risk factor for the incidence of anemia.^8^

Thus, given the need to contribute to the body of scientific evidence on the relationship between sleep duration and iron deficiency anemia, this article aimed to investigate the association between iron deficiency anemia and sleep duration in the first year of life. To the best of our knowledge, this is the first study with this approach developed in the first year of life, and our main hypothesis was that infants with iron deficiency anemia in the first 6 months of age would have short sleep duration in the first year of life.

## METHOD

The data used for this study come from the birth cohort: “*SAND* — Health, Food, Nutrition, and Child Development: a cohort study”, which aimed to analyze aspects related to health, food, nutrition, and the development of infants from birth until the first year of life. The research, carried out in Rio Largo, the third largest municipality in the state of Alagoas, located 28 km away from the capital Maceió, was approved by the Research Ethics Committee, and all mothers signed an informed consent form. Methodological details are described in the investigation by Melo et al.^9^

Between February 2017 and August 2018, data collections were carried out four times as follows:

1. Birth of the child (maximum seven days after birth);
2. At 3 months of age;
3. At 6 months of age; and
4. At 12 months of age.

Teams of previously trained researchers applied structured and precoded questionnaires to obtain social, economic, demographic, and health information about mothers and infants. Information on the child’s weight and length at birth was obtained through consultation of medical records.

Of the total number of births that occurred in this period (n=394), 109 dyads did not meet the eligibility criteria, 41 refused to participate in the research, and 1 mother was identified with a language disorder that compromised communication, totaling 243 infants at baseline. “Follow-up losses” were considered when the mother-child pair was not located in two consecutive follow-up periods, when they moved to other cities, or when mothers no longer had a regular bond/coexistence with their children, as in cases of donation for adoption. Thus, at 3 months, 210 children participated in the research; at 6 months, 198; and at 12 months, 186 infants. Given the purpose of our study to investigate the association between sleep duration and iron deficiency anemia, our analyses were conducted with 123 infants, who had complete data on these variables.

Sleep information was obtained at 3, 6, and 12 months of age, through the validated and translated version into Brazilian Portuguese of the semistructured Brief Infant Sleep Questionnaire (BISQ), which investigates the usual and last week’s sleep characteristics of the child, according to mothers’ reports.^10^

The measures used in this study were as follows:

1. Nighttime sleep duration (from 7 p.m. to 7 a.m.) and
2. Daytime sleep duration (from 7 a.m. to 7 p.m.).

Total sleep duration was represented by the sum of hours slept during the night and the day. To obtain the average daily sleep time in the first year of life, the arithmetic mean of sleep duration at 3, 6, and 12 months was calculated. The cutoff point for classifying short sleep duration was total sleep time <10 h/day.^1^

Infants underwent biochemical tests at birth and at 6 months of age in a clinical analysis laboratory enrolled in the National Quality Control Program. Blood samples were collected by venipuncture between 7 a.m. and 10 a.m., with fasting for at least 3 h, within 1 week after birth and, later, at 6 months of age, prioritizing a window of at most 3 days before/after the exact day of the child’s birth. The cutoff point for anemia was hemoglobin value <11.0 g/dL.^11^

Within 24 h after collection, data were double-entered and validated in the Epi Info software (Version 3.5.4). Analyses were performed using the Stata/SE 15.1 software (Stata Corp LP, College Station, TX, USA).

For descriptive statistics, absolute (%) and relative frequencies were calculated, and the categorical variables were compared using the chi-square test. The comparison of sleep duration average between infants with and without anemia was performed using the Student’s t-test.

To verify the association between sleep duration (dependent variable) and iron deficiency anemia (independent variable), logistic regression models were used, estimating odds ratios (ORs) and 95% confidence intervals (CIs). Infants were categorized into two groups according to sleep duration: the not short-sleepers group (≥10 h at 3, 6, and 12 months of age) and the short-sleepers group (<10 h at 3, 6, and/or 12 months of age). Anemia groups were defined as follows: with anemia group (hemoglobin <11 g/dL at birth and/or at 6 months of age) and without anemia group (hemoglobin ≥11 g/dL at birth and at 6 months of age).

Linear regression models were used to verify the association between hemoglobin values at 6 months and mean sleep time from 3 to 12 months of age.

In the analysis, the following variables were explored as potential confounders: family income (continuous: Brazilian reals [BRL]), maternal schooling (categorical: ≤8 or >8 years), gender, and body mass index (BMI)-for-age (continuous: z-scores) at birth, selected for their associations with both anemia and sleep.^4,12,13^ A p-value <0.05 was adopted for all analyses to define statistical significance.

## RESULTS

Sociodemographic, gestational, perinatal, and infant characteristics of the 123 participants (52.03% female), concerning the presence of short sleep duration, are presented in Table 1. It is verified that almost half (∼43%) of the families received less than one minimum wage, and ∼38% were enrolled in an income transfer program. Regarding the mothers, more than a third had less than 8 years of schooling and was adolescents, ∼60% were multiparous, and ∼85% lived with their partners. A total of ∼37% of infants were short sleepers (<10 h) from the third to 12th month of life, and more than 85% were anemic (Hb <11 g/dL) in the first semester of life, with this prevalence being higher among short sleepers (93.33%), when compared to the group of infants without short sleep duration (80.77%) (p≤0.05).

**Table 1.** Sociodemographic, gestational, perinatal, and infant characteristics according to sleep duration.

	Sleep duration*			
	All n (%)	Non-short sleepers n (%)	Short sleepers n (%)	p-value^†^
Total	123 (100.0)	78 (63.4)	45 (36.6)	
Socioeconomic and demographic				
Family income, ≤1 MW^‡^	53 (43.1)	32 (41.0)	21 (46.7)	0.11
Income transfer program, yes	48 (39.0)	29 (37.2)	19 (42.2)	0.58
Mother’s skin color, white	18 (14.6)	10 (12.8)	8 (17.8)	0.48
Maternal schooling, ≤8 years	44 (35.8)	23 (29.5)	21 (46.5)	**0.05**
Lives with partner, yes	105 (85.4)	66 (84.6)	39 (86.7)	0.75
Maternal age, ≤19 years	39 (31.7)	24 (30.8)	15 (33.3)	0.76
Gestational and perinatal				
Primiparous, yes	49 (40.8)	33 (42.9)	16 (37.7)	0.54
Prenatal, ≥6 consultations	68 (55.3)	41 (52.6)	27 (60.0)	0.42
Infants				
Gender, female	64 (52.0)	38 (48.7)	26 (57.8)	0.33
Birth weight, ≥2,500 g	118 (95.9)	73 (93.6)	123 (100.0)	0.08
Breastfed at 3 months, yes	97 (88.2)	63 (88.7)	34 (87.2)	0.80
Breastfed at 6 months, yes	82 (69.5)	52 (69.3)	30 (69.8)	0.96
Breastfed at 12 months, yes	71 (58.7)	43 (55.1)	28 (65.1)	0.28
Anemia at birth, yes	13 (11.40)	6 (8.5)	7 (16.3)	0.20
Anemia at 6 months, yes	99 (84.6)	61 (80.3)	38 (92.7)	0.07
Anemia at birth and/or 6 months, yes	105 (85.4)	63 (80.8)	42 (93.3)	**0.05**

MW: minimum wage. *Sleep duration groups were defined as follows: not short-sleep group ≥10 h at 3, 6, and 12 months of age and short-sleep group <10 h at 3, 6, and/or 12 months of age; ^†^p-values are derived from the chi-square test, and significant values ≤0.05 are shown in bold; ^‡^Refers to the minimum wage in the year of study (2017): BRL 950.00.

Table 2 shows that, after adjusting for potential confounding variables (family income, maternal schooling, gender, and BMI at birth), infants with iron deficiency anemia in the first semester had higher odds of being short sleepers at some point during the first year of life (OR: 4.02, 95%CI 1.02–15.76, p=0.04).

**Table 2.** Associations between iron deficiency anemia* and short sleep duration.^†^ Data are presented as OR (95%CI) for the anemia group in comparison to those without the anemia group. Logistic regression models are adjusted for family income, education, gender, and birth body mass index.

	OR (95%CI)	p-value
Unadjusted model	3.33 (0.90–2.22)	0.06
Adjusted model	4.02 (1.02–15.76)	0.04

BMI: body mass index; OR: odds ratio; CI: confidence interval. *Anemia groups were defined as follows: with anemia group: hemoglobin <11 g/dL at birth and/or at 6 months of age and without anemia group: hemoglobin ≥11 g/dL at birth and at 6 months of age; ^†^Sleep duration groups were defined as follows: not short-sleep group ≥10 h at 3, 6, and 12 months of age and short-sleep group <10 h at 3, 6, and/or 12 months of age.

**Figure 1.**
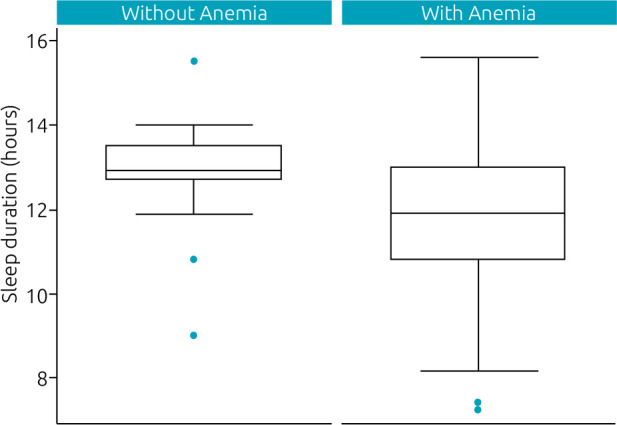
Box plot graph comparing sleep duration* in infants with and without iron deficiency anemia^2^.

In the box plot graph (Figure 1), it is observed that the group with anemia in the first semester had a significantly (p=0.02) shorter average duration of sleep (11.83±1.67) compared to the group without anemia (12.78±1.34).

*Sleep duration refers to the average time (hours) at 3, 6, and 12 months of age; ^†^Anemia groups were defined as follows: with anemia group: hemoglobin <11 g/dL at birth and/or at 6 months of age and without anemia group: hemoglobin ≥11 g/dL at birth and at 6 months of age. p-value (0.02) is derived from the Student’s t-test.

**Figure 2.**
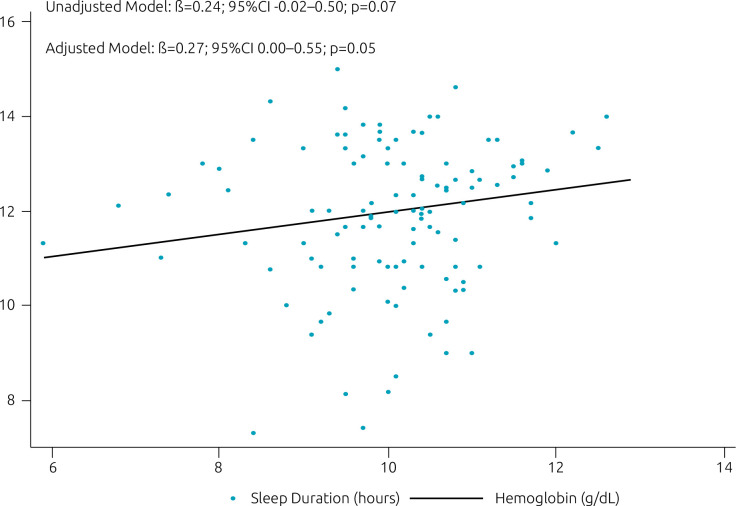
Association between hemoglobin values* and sleep duration.^†^ Linear regression model is adjusted for family income, education, gender, and birth body mass index.

Figure 2 shows that the higher the hemoglobin levels in the sixth month, the greater the average total sleep time in the first year of life. This linearity, adjusted for confounding variables, demonstrated that each additional unit of hemoglobin increased sleep time by 16.2 min (β: 0.27, 95%CI 0.00 to 0.55, p=0.05).

*Hemoglobin values at 3 months of age; ^†^Sleep duration refers to the average time (hours) at 3, 6, and 12 months of age.

## DISCUSSION

Our study focused on the relationship between iron deficiency anemia in the first 6 months of age and short sleep duration from 3 to 12 months of age. As far as we know, this is the first study to investigate the association between iron deficiency anemia and sleep time during the first year of life, with few studies worldwide on the subject, which reinforces the contribution of our findings to the understanding advancement of the interrelationship of these conditions.

Our main findings were that infants with iron deficiency anemia in the first semester of life were 40% more likely to have short sleep duration at some point between 3 and 12 months, and for each unit increase in the values of hemoglobin, sleep time increased by 16.2 min. These associations were independent of family income, maternal schooling, gender, and BMI at birth. In addition, the average duration of sleep was significantly shorter among infants with anemia in the first semester (11.83±1.67 h) when compared to those without deficiency in this period (12.78±1.34 h).

It is worth mentioning the high prevalence of anemia in the first semester of life (85%) is higher than that observed in national surveys and is characterized as a serious public health problem (above 40%), according to criteria defined by the World Health Organization (WHO).^11^ Data from the National Demographic and Health Survey^14^ identified a prevalence of 20.9 and 25.5% in Brazil and the Northeast region, respectively, among children under 5 years of age, and recent data from the National Child Food and Nutrition Study showed a national prevalence of 10.0% in children under 5 months and 18.9% from 6 to 23 months.^15^

Regarding sleep time, although the averages verified between the 3^rd^ and 12^th^ months of age are within the range considered appropriate (10–18 h),^1^ 36.59% of the infants were short sleepers (<10 h). Data from two representative population cohorts in Finland, CHILD-SLEEP and FinnBrain, showed that children had a mean sleep time of 13.7 h at 6 months of age and 12.8 h at 12 months of age.^16^

Although there are no national sleep surveys in Brazil, data from a cross-sectional population-based study conducted in the five Brazilian regions (North, Northeast, Midwest, Southeast, and South), including 350 children aged from 0 to 3 years, corroborate our findings. This study identified that the average duration of sleep was below or within the lower limit of the recommended range, with a prevalence of sleep problems of 20% in the first 3 years of life.^17^

A cohort in the city of Pelotas investigated the sleep duration of 3,824 children aged between 3 and 48 months and found that 9.1% slept less than recommended, with the risk being higher among children with mothers with low education and with alcohol consumption up to 3 months after delivery.^18^

Regarding the association between anemia and short sleep duration evidenced in our analyses, our findings corroborate similar studies. In a study conducted by Kordas et al.^6^ on the island of Pemba in Zanzibar, with 684 children aged from 6 to 18 months, the prevalence of short sleep duration among the group with anemia was higher than that observed in the control group, equivalent to 41.2 and 29.3%, respectively.

Although the relationship between anemia and sleep is not fully elucidated, some mechanisms are proposed to justify it. Rao et al^19^ and Connor and Menzies^20^ demonstrated that iron is an essential component for the metabolism of neurotransmitters, being a cofactor for the enzymes tryptophan hydroxylase and tyrosine hydroxylase, which act, respectively, in the synthesis of serotonin and dopamine, which plays a role in the regulation of the sleep-wake cycle.^21^ In addition, a study demonstrated that the levels of dopamine D1 and D2 receptors were decreased in rats that had iron deficiency anemia.^22^

Peirano et al.,^8^ analyzing longitudinally associations between iron deficiency anemia in childhood and sleep disorders, observed that children with iron deficiency anemia in the first 2 years of life, compared to those without iron deficiency in this period, had different sleep patterns at 4 years of age. The group anemia group showed a longer duration of rapid eye movement (REM) sleep in the first third of the night and shorter in the last third, in addition to a shorter latency time for REM sleep and a shorter non-REM sleep stage, also having more episodes of REM sleep in the first third of the night and fewer episodes in the second third. The study also showed that, even when undergoing treatment with iron supplementation and not presenting anemia at age of 4 months, the children presented structural changes in sleep.

Corroborating this finding, on the long-term effects caused by iron deficiency on sleep, another study, with adults from the Kailuan Study cohort, including 12,614 Chinese, longitudinally investigated the association between iron deficiency anemia and insomnia and found that individuals with anemia in 2006–2007 were more likely to have insomnia 6 years later (2012) compared with individuals without prior anemia.^7^

Such studies suggest, therefore, that the impact of anemia on sleep duration, structure, and patterns occurs in the short, medium, and long term, lasting in subsequent years and adding the inherent consequences of sleep deprivation and poor sleep quality both in development and in physical and mental health.

Although our hypothesis assumed hemoglobin levels and anemia as predictors of sleep time and short sleep duration, it is worth mentioning that recently, although few and restricted to adults, studies in the literature propose bidirectional associations between these variables, demonstrating that sleep duration can also represent an increased risk of incidence of iron deficiency anemia.

A prospective study conducted in China with 84,791 participants aged between 18 and 98 years, of whom 2698 developed anemia, aimed to assess the impact of short sleep duration on anemia. In the fragmented analysis of age groups, the authors found that individuals under 60 years of age and who had a sleep duration of ≤5 h were more likely to develop anemia, with the risk being 1.24 times greater when compared to the control group.^23^

In any case, we must bear in mind that both insufficient sleep and iron deficiency anemia individually represent risk factors for several unfavorable health outcomes. In our population, in addition to the high prevalence of both conditions, the hypothesis was confirmed about the relationship between anemia and sleep duration in the first year of life.

Finally, our study has some strengths and limitations. First, we emphasize that we used data from the first and only birth cohort in the state of Alagoas (Brazil). In addition, we conducted interviews at fixed times (at 6 and 12 months postpartum) to minimize age variability among children. Although more accurate information on sleep behavior can be obtained through objective measurements, there is evidence that the mother’s reports are consistent with actigraphy measurements. Finally, since the scale used in the present study (BISQ) is characterized as a psychometric tool, clinical and ecological support for clinical and research purposes are widely used in international studies.^24^

Regarding limitations, it is worth noting that the infants studied represent the population of a vulnerable region of Brazil; therefore, the interpretations of the results must be considered with caution in populations with different socioeconomic characteristics.

In conclusion, our results confirmed our hypothesis that iron deficiency anemia is associated with short sleep duration in the first year of life, regardless of family income, maternal schooling, gender, and BMI at birth. Given the high percentage of infants with short sleep duration and the consequences it can have on child growth and development, it is suggested, in the context of public health, the use of subjective tools to measure child sleep duration and quality in pediatric clinical practice and routine primary care protocols, in order to identify and intervene early on sleep-related problems, advising parents on the importance of sleep hygiene. In addition, as it is a subject that has been little investigated in the scientific literature, we suggest further studies, especially longitudinal investigations with longer follow-ups to verify the impact of anemia on sleep duration and quality at subsequent ages.

## Data Availability

The database that originated the article is available with the corresponding author.
